# Bilateral sternalis muscle in a Sudanese cadaver

**DOI:** 10.1016/j.ijscr.2021.106511

**Published:** 2021-10-16

**Authors:** Khalid A. Awad, Ghassan E. Ahmed, Qabas A.Allah A.llah, Hayat A. Ahmed

**Affiliations:** a Department of Anatomy, Faculty of Medicine, University of Khartoum, Sudan; b SAMER Research Group, MBBS Student, Faculty of Medicine, University of Khartoum, Khartoum, Sudan

**Keywords:** Episternalis, Presternalis, Sternalis brutorum, Rectus thoracic, Rectus sterni, Superficial rectus sterni

## Abstract

**Introduction and importance:**

Sternalis/rectus sterni is a rare muscle found in the anterior chest wall, it occurs in 35% of humans. The early detection of its presence is critical in regular mammogram screening in order to avoid possible differential diagnostic dilemma.

**Case presentation:**

We report here a case of the sternalis muscle observed bilaterally, it was found during routine dissection session of an elderly male cadaver in the dissection room, Faculty of Medicine, University of Khartoum.

**Clinical discussion and conclusion:**

Sternalis muscle is a familiar entity to anatomists, but can pose a diagnostic and surgical dilemma to some clinicians. Presence of the muscle can be confusing in regular mammogram screening and CT and MRI should be utilized to clear the dilemma, and further evidence needs to be explored and studied.

## Highlights

• Sternalis/rectus sterni is a rare muscle found in the anterior chest wall, it occurs in 3-5% of humans.
• We report here a case of the sternalis muscle observed bilaterally, it was found during a routine dissection session of an elderly male cadaver in the dissection room, Faculty of Medicine, University of Khartoum.
• Presence of the muscle can be confusing in regular mammogram screening and CT and MRI should be utilized to clear the dilemma, and further evidence needs to be explored and studied.
• Considering its insignificant function, it could be used as a muscle flap in surgical reconstructions of the head, neck, chest wall and breast.

## Abbreviations

SM: Sternalis Muscle
CT: Computed Tomography
MRI: Magnetic Resonance Imaging

## 1 Introduction

Sternalis muscle is a rare anatomic variant of the chest wall musculature, it is found in front of the sternal end of the pectoralis major parallel to the margin of the sternum. It is supplied by the anterior thoracic nerves and is probably a misplaced part of the pectoralis [1]. In the literature it is also known as episternalis, presternalis, sternalis brutorum, rectus thoracic, rectus sterni and superficial rectus sterni [2].

Bartolemen Cabrolio first noticed this muscle in the year 1604 and a proper description was first made by Du Puy in 1726, The occasional presence of the Sternalis muscle in human has always been a subject of great interest to anatomists [3].

This muscle occurs in 3–5% of humans, found slightly more frequently in females than males [3], The incidence of the sternalis muscle varies widely between ethnic groups. Its reported incidence is 4% to 7% in the white population, 8.4% in the black population, and 11.5% in the Asian population [4].

This variant muscle is a dilemma for surgeons and radiologists whereas a matter of interest for anatomists. The literature of the subject provides constantly varying assessments of the morphological presentation of this muscle, especially in its nerve supply, origin, and its tendency to present bilaterally or unilaterally [5].

The early detection of its presence is critical in regular mammogram screening in order to avoid possible differential diagnostic dilemma. Additionally, there are potential surgical benefits, as it can be used as a flap in reconstruction surgery of the head and neck, anterior chest wall, and breast [5]. We report here a case of the sternalis muscle observed bilaterally in a Sudanese cadaver.

This case report has been reported using SCARE guidelines for surgical case reports [6].

## 2 Case presentation

Sternalis muscle was found during routine dissection session of an elderly male cadaver in the dissection room, Faculty of Medicine, University of Khartoum.

**Fig. 1:**
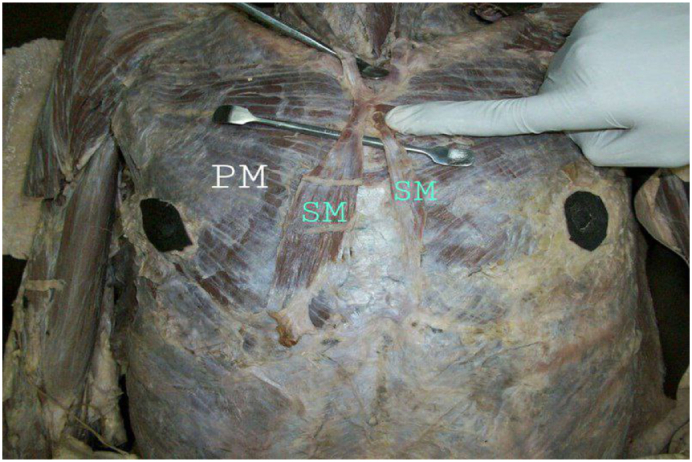
Shows bilateral Sternalis muscle seen overlying pectoralis major muscle. (PM: pectoralis muscle, SM: Sternalis muscle).

Two muscles were bilaterally positioned, distinct, 9 cm long in the left hemithorax, and 14 cm long in the right hemithorax. They were covered by superficial fascia and located anteriorly and obliquely to the pectoralis major muscle (Fig. 1).

**Fig. 2:**
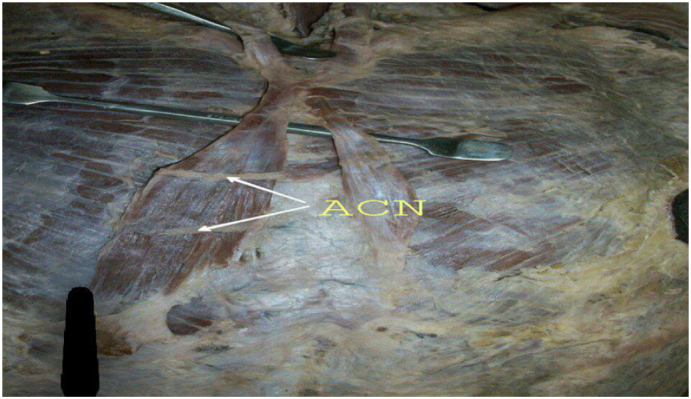
Shows two anterior cutaneous nerves seen crossing in front of the right sternalis muscle. (ACN: anterior cutaneous nerve).

On the right side, the muscle attached to the lower end of the fifth costal cartilage. Its cranial end blended with the sternal head of the right sternocleidomastoid muscle. The right one was broader than the left and had two anterior cutaneous nerves seen crossing in front (Fig. 1, Fig. 2).

On the left side the muscle attached to the upper part of the fifth costal cartilage and terminates cranially in the pectoral fascia, approximately at the sternal angle. It was slender than the right one (Fig. 1, Fig. 2).

The two muscles were easily separable from the underlying pectoralis major muscle. Pectoralis major and minor muscles were normally placed.

## 3 Discussion

The sternalis muscle is an unusual normal variant that is familiar to anatomists, but can represent a diagnostic dilemma to clinicians in terms of differentiation from pathology [7]. It could be unilateral or - as with our case- bilateral, and it is twice as often unilateral as bilateral [1]. The muscle looks like a slender band of fibers lying superficial to the pectoral fascia and running parallel to the sternum. The fibers were described to arise from the rectus abdominis sheath and terminate upon the pectoralis fascia, upper sternum, sternum, costal cartilages or the medial heads of the sternocleidomastoid muscle [8], [9]. It was first seen on mammography of six women during screening and diagnostic mammographic imaging of 32,000 women [7].

Incidence of the sternalis muscle varies among populations, ranging from 4 to 7% among the white population, 8.4% among Africans, and 11.5% among Asians [10]. Several theories were developed regarding the origin of the muscle, either claimed as a remnant of the panniculus carnosus [11], a vestige of the cuticular muscle of mammals and presents in the form of axillary arch [12], or described as a part of a ventral, longitudinal column of muscles arising at the ventral tips of the hypomeres [13]. Though its function is still unknown, but it is thought to participate in the shoulder joint movement or have a role in the elevation of the lower chest wall [14].

Regarding the nerve supply, two sets of nerves are closely related to the sternalis muscle: the pectoral nerves [15], and the anterior cutaneous branches of the intercostal nerve [11]. It was also reported that in 55% of the cases it was innervated from the external and internal thoracic nerves [16].

No clinical symptoms were found to be associated with the existence of the sternalis muscle, but it may present alterations in the ECG [9], or confused as a malignant chest wall lesion, such as breast carcinoma or hematoma. This confusion can be settled by utilization of CT and MRI imaging [14]. It is important to be aware of such entity in anterior thoracic wall surgical dissection and particularly in breast, and cardiothoracic surgery. Considering its insignificant function, it could be used as a muscle flap in surgical reconstructions of the head, neck, chest wall and breast [17], [18], [19], [20], [21].

## 4 Conclusion

The sternalis muscle is an anatomical chest wall variant, with uncertain origin and function. It is a familiar entity to anatomists, but can pose a diagnostic and surgical dilemma to some clinicians. Presence of the muscle can be confusing in regular mammogram screening and CT and MRI should be utilized to clear the dilemma, and further evidence needs to be explored and studied.

## Ethical approval and consent

The Cadaver was an unclaimed body, was taken for educational purposes with permission and agreement between Forensic unit – Federal ministry of Health and Anatomy Department – Faculty of Medicine, University of Khartoum. During educational dissection, Cadavers were treated according to the ethical guidelines of University of Khartoum, and during the preparations we discovered the existence of the rare Sternalis muscle and decided to report it. The anatomy department can be considered the legal guardian.

## Sources of funding

No funding was received for the work involved in this article.

## CRediT authorship contribution statement

KA: Supervisor, assistant professor of Anatomy, has supervised, written and revised this case report and managed the dissection of the cadaver.

GA, QA, HA: Participated in manuscript writing and revision.

All authors read and approved the final manuscript.

## Provenance and peer review

Not commissioned, externally peer-reviewed.

## Declaration of competing interest

The authors report no conflicts of interests.
